# Reevaluating Donor-recipient Sex Mismatch and Survival After Liver Transplantation: A Contemporary Cohort Study

**DOI:** 10.1097/TXD.0000000000001968

**Published:** 2026-06-18

**Authors:** Nicola Sariye Pollmann, Catherine Parmentier, Falk Rauchfuß, Utz Settmacher, Lukas Pollmann, Markus Selzner

**Affiliations:** 1 Department of General, Visceral, and Vascular Surgery, Jena University Hospital, Jena, Germany.; 2 Ajmera Transplant Centre, Toronto General Hospital, University Health Network, Toronto, ON, Canada.

## Abstract

**Background.:**

Identifying an optimally matched donor is a critical determinant of outcomes following liver transplantation (LT). Several donor- and recipient-related factors have been shown to influence posttransplant survival. However, the impact of donor-recipient sex mismatch (DRSM) on long-term outcomes remains controversial, with inconsistent findings reported across prior studies. This study aimed to further clarify the contemporary role of DRSM in LT outcomes.

**Methods.:**

We conducted a single-center retrospective cohort study including 1146 adult patients who underwent LT at the Ajmera Transplant Centre, Toronto, between January 2014 and January 2022. Donor and recipient characteristics, including age, sex, and donor type, were analyzed. The primary outcome was patient survival up to 10 y of follow-up. Comparative analyses were performed between recipients with mismatch (DRSM+) and those without (DRSM–), as well as relevant subgroup analyses.

**Results.:**

In unadjusted survival analysis, recipients in the DRSM+ group demonstrated slightly higher survival compared with DRSM– recipients up to 10 y following LT (*P* = 0.018). However, after multivariable adjustment and propensity score matching, survival was comparable between groups, and DRSM was not identified as an independent predictor of mortality. Subgroup analyses showed no adverse effect of DRSM in deceased donor transplantation (*P* = 0.547), while a survival difference was observed in the living donor cohort (*P* < 0.001). In addition, female recipients exhibited improved survival compared with male recipients, irrespective of DRSM.

**Conclusions.:**

DRSM does not appear to be an independent risk factor for reduced long-term survival after LT. Moreover, the composition of favorable recipient and donor characteristics seems to be the main factors influencing long-term survival. These findings suggest that DRSM alone should not preclude donor selection in clinical practice.

## Graphical abstract

**Figure:**
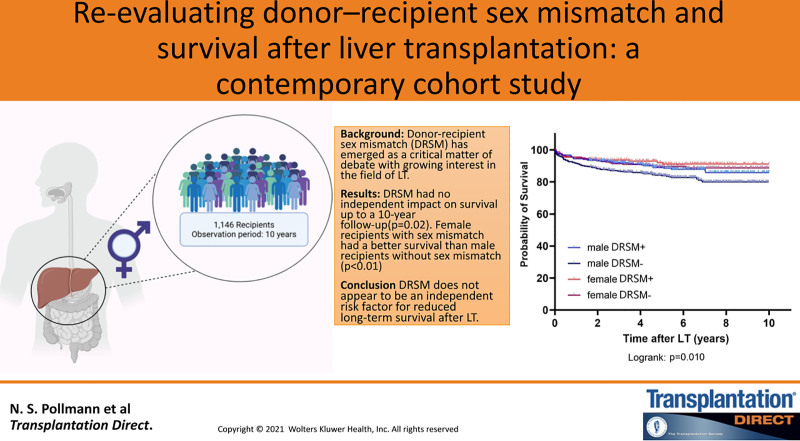

## INTRODUCTION

The persistent global shortage of donor organs presents a significant challenge in the field of liver transplantation (LT). As the demand for suitable donors continues to grow, the process of donor-recipient matching becomes increasingly complex. Within this framework, it is crucial to determine whether the selection criteria for both donors and recipients should be well aligned to ensure successful transplantation outcomes.^1^ However, in the context of LT, donor-recipient sex mismatch (DRSM) is generally not considered an independent determinant when evaluating a potential liver graft. Nevertheless, sex-related differences may indirectly influence donor selection through factors such as graft size compatibility or donor characteristics, which are important considerations during organ acceptance.^2^

In this view, sex-related disparities in LT represent an important aspect of donor and recipient matching because biological and anatomical differences between male and female donors and recipients may influence both access to transplantation and posttransplant outcomes.^3-5^ Since the introduction of the Model for End-Stage Liver Disease (MELD) score in 2002, women have been reported to experience a higher risk of mortality or removal from the transplant waiting list compared with men.^6^ Moreover, data reveal that women who are regularly listed considering MELD without exceptional points because of hepatocellular carcinoma are less likely to be ranked first for a match and show an increased risk of hospitalization compared with men.^7,8^ Although women are less likely than men to receive LTs, they are more frequently represented as liver donors.^3,6^ These observations may reflect a combination of biological, anatomical, and sociocultural factors, highlighting the complex role of sex-related differences in transplantation practice.^3^

Given these disparities, the question arises as to whether compatibility between donor and recipient sex influences transplantation outcomes.^9^ Differences in body size, liver volume, and hormonal and immunological characteristics between males and females have led to the hypothesis that DRSM could affect graft function and long-term survival after LT.^4^ Consequently, DRSM has been repeatedly investigated as a potential determinant of posttransplant outcomes.^6^ Early studies suggested that certain DRSM combinations, particularly female-to-male transplantation, might be associated with differences in graft survival.^9,10^ These findings were supported by several subsequent investigations and meta-analyses suggesting that biological differences between donors and recipients may influence transplant outcomes.^2,11^ However, more recent registry-based analyses and contemporary cohort studies have produced heterogeneous results, and several reports have suggested that the apparent impact of sex mismatch may diminish after adjustment for donor and recipient characteristics.^12,13^ Consequently, the independent role of DRSM in LT outcomes remains a matter of debate.^5,14^

In this context, contemporary cohort studies with detailed clinical data remain important to reassess the relevance of DRSM within modern transplantation practice. The present study, therefore, aimed to reevaluate the impact of DRSM on long-term survival after LT in a contemporary cohort from a high-volume transplant center.

## MATERIALS AND METHODS

### Study Characteristics

This retrospective, single-center, observational study aimed to assess the impact of DRSM on the outcome of adult transplant recipients who underwent LT between 2014 and 2022 at the Ajmera Transplant Center at Toronto General Hospital in Toronto, Ontario, Canada. We recruited all recipients who underwent first-time deceased donor (DD) and living donor (LD) LT. The observation period ended in 2024, providing a minimum follow-up period of 24 mo. Patients included in the study received LTs exclusively.

Before analysis, all data were anonymized. Because of the retrospective design and use of de-identified data, informed consent was waived. Furthermore, all procedures followed were in accordance with the ethical standards of the responsible committee on human experimentation (institutional and national) and the Helsinki Declaration of 1975, as revised in 2008. This study was approved by the local ethics committee (20-5877.4 REB) and registered at ClinicalTrials.gov (NCT07250919).

Two study populations were defined: LT recipients with a mismatched donor (DRSM+) and LT recipients with a matched donor (DRSM–). For each liver recipient, the demographic characteristics, cause of liver disease, type of donation, and MELD score were recorded. Donor information was obtained from the Trillium Gift of Life Network (Ontario Health) and the local database for living donations.

The dataset was subsequently screened for missing information. Cases were excluded if >50% of the data were missing. The variables used in this study did not exceed 30% missing values.

The primary endpoint was overall survival. A comparison was made between the 2 study groups: DRSM+ and DRSM–. Furthermore, male and female recipients were analyzed separately, resulting in 4 comparisons: male-to-male, female-to-male, male-to-female, and female-to-female.

### Statistical Analysis

Group comparisons were conducted using the Mann-Whitney *U* test for nonnormal data, Fisher exact test for categorical variables, and Student *t* test for normal data. In instances where the continuous variables were found to be normally distributed, as determined by the Kolmogorov-Smirnov test, the mean and SD were presented. Categorical variables are presented as numbers (n) and percentages (%). Continuous variables are presented as median and interquartile range.

**FIGURE 1.**
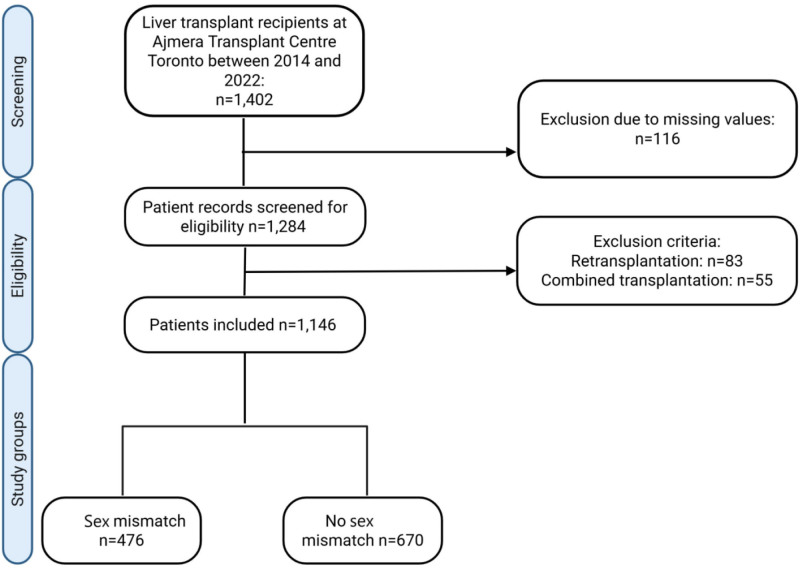
Number of liver transplant recipients meeting eligibility criteria for study inclusion between 2014 and 2022 at the Ajmera Transplant Centre in Toronto.

The comparison of patient survival between DRSM+ and DRSM– was estimated using the Kaplan-Meier method and the log-rank test (for *P* ≤ 0.05). Statistical tests were conducted using MedCalc Software (MedCalc Software Ltd., Ostend, Belgium), and the results were visualized using GraphPad Software (Version 10.4.0; GraphPad Software, Inc., San Diego, CA). The graphical abstract and Figure 1 were created using BioRender (BioRender, Toronto, ON, Canada).

Multivariable Cox regression analysis and propensity score matching were performed to minimize the confounding effects related to donor and recipient characteristics. Propensity score matching was performed to compare the DRSM+ and DRSM– groups. In particular, the case-control tool MedCalc software (MedCalc Software Ltd.) was used, using a greedy caliper propensity score algorithm. Four variables—donor age, recipient age, type of donation, and donor sex—were selected for matching. The selection of the variables, donor sex, and type of donation was made because of a significant difference in DRSM+ and DRSM– (*P* < 0.05). A range of 2 y was selected for age matching, while exact matching was mandatory for the remaining variables. Subsequently, patient survival of the matched groups of DRSM+ and DRSM– was compared using the Kaplan-Meier method and the log-rank test (for *P* ≤ 0.05).

To explore the independent risk factors for patient survival, both univariable and multivariable Cox regression models were fitted. The baseline characteristics of the donors and recipients were selected for analysis, including donor age, body mass index (BMI), donation type, sex, recipient MELD score, age, BMI, and sex. Hazard ratios (HRs) and 95% confidence intervals (CIs) were calculated. The proportional hazards assumption was assessed graphically.

A subgroup analysis of DRSM was conducted using the Kaplan-Meier method to compare the long-term survival disparities between female and male recipients. Furthermore, baseline comparisons of the DRSM+ and DRSM– groups were conducted using the Mann-Whitney *U* test for nonnormally distributed data, the chi-square test for categorical variables, and Student *t* test for normally distributed data.

## RESULTS

### Study Cohort

The present study included 1146 patients who underwent LT between 2014 and 2022 and were subjected to minimal follow-up for 24 mo postsurgery (Figure 1). The median follow-up period was 6 y (95% CI, 5-6 y) in both the DRSM+ and DRSM– groups. Of these, 670 recipients were part of the DRSM– group, whereas 476 recipients were part of the DRSM+ group. A comparative analysis of baseline donor and recipient characteristics showed that the DRSM+ and DRSM– groups were comparable across several variables, although differences in donor and recipient sex distributions as well as the type of donation were observed. Recipients in the DRSM+ and DRSM– groups exhibited similar BMI, MELD, and age. Donors of the DRSM+ and DRSM– groups were of similar age and had a comparable BMI (Table 1). Analysis of the whole study cohort showed that only 29% (n = 412) of recipients were female, and 59% (n = 834) of donors were female.

**TABLE 1.** Baseline characteristics of LT recipients and donors

Patient cohort	DRSM– (n = 670)	DRSM+ (n = 476)	*P*
Recipient characteristics			
Age, mean ± SD, y	56.8 ± 10.5	56.4 ± 11.8	0.622
BMI, mean ± SD, kg/m^2^	28.2 ± 5.9	27.8 ± 15.1	0.718
Sex, female, n (%)	235/670 (35)	177/476 (37)	0.492
MELD score, mean ± SD	19.7 ± 9.6	19.3 ± 9.4	0.527
Donor characteristics			
Age, mean ± SD, y	43.9 ± 16.1	44.9 ± 15.6	0.283
BMI, mean ± SD, kg/m^2^	26.8 ± 5.4	26.7 ± 5.5	0.713
Sex, female, n (%)	535/670 (58)	299/476 (63)	<0.001
Type of donation, n (%)			0.016
Donation after brainstem death	374/670 (56)	233/476 (49)	
Donation after circulatory death	115/670 (17)	77/476 (16)	
Living donor transplantation	181/670 (27)	166/476 (35)	
Other^^*a*^^	42 (6)	26 (6)	

^*a*^Split liver grafts from deceased donors.

BMI, body mass index; DRSM, donor-recipient sex mismatch; LT, liver transplantation; MELD, Model for End-Stage Liver Disease.

In addition, hepatitis C virus infection was the most prevalent cause of liver disease in the DRSM+ and DRSM– groups, accounting for 20% and 24% of cases, respectively. The second most prevalent diagnosis in both groups was ethyl toxic-related cirrhosis, accounting for 20% of the cases. Furthermore, there were differences in the distribution of females and males across donors and recipients. The recipients of the DRSM+ and DRSM– groups exhibited a comparable sex distribution. In contrast, among donors, a significantly higher number of females was observed in the DRSM+ group than in the DRSM– group (*P* < 0.001; Table 1).

### Overall Survival and Risk Factors for Mortality After LT

**FIGURE 2.**
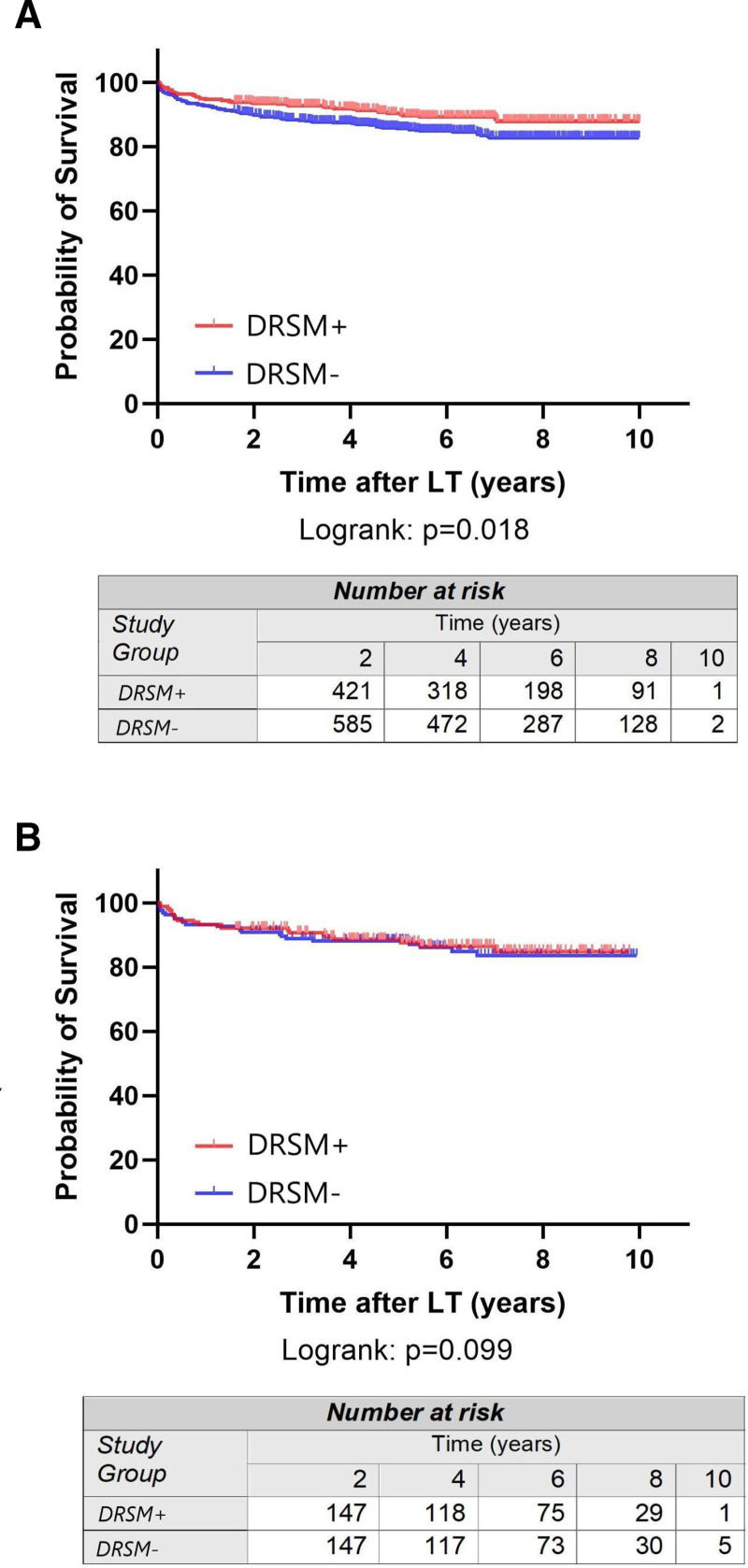
Overall patient survival of LT recipients. DRSM+ and DRSM− donors (red and blue) are shown for all unmatched recipients in (A), and survival of recipients after matching risk factors by propensity score matching is compared in (B). Follow-up in years, up to 10 y. *P* value as log-rank. Probability of survival in percent. Patients at risk are given. Loss of follow-up is censored. DRSM, donor-recipient sex mismatch; LT, liver transplantation.

Over a maximum follow-up of 10 y, recipients in the DRSM+ group demonstrated slightly higher survival compared with recipients in the DRSM– group in the unadjusted Kaplan-Meier analysis (*P* = 0.018). The overall survival rates in the DRSM+ group at 2, 5, and 10 y were 94%, 89%, and 88%, respectively, compared with 90%, 86%, and 83% in the DRSM– group (Figure 2A).

To account for potential differences in donor and recipient characteristics between groups, propensity score matching was performed based on donor age, recipient age, donor sex, and type of donation. The matched DRSM+ and DRSM– cohorts each included 164 recipients. After matching, survival probabilities were comparable between groups, with no significant differences observed at 2, 5, or 10 y after transplantation (Figure 2B).

Similarly, multivariable Cox regression analysis did not identify DRSM as an independent predictor of long-term survival after LT (Table 2).

**TABLE 2.** Univariable and multivariable Cox regression model

Patient cohort	Univariable		Multivariable	
	HR (95% CI)	*P*	HR (95% CI)	*P*
Donor age	1.02 (1.01-1.03)	<0.001	1.01 (1.00-1.03)	0.030
Donor BMI	1.00 (0.96-1.03)	NS	0.99 (0.95-1.03)	NS
Donor type DBD vs DCD	0.71 (0.44-1.14)	NS	0.83 (0.49-1.40)	NS
Donor type LD vs DBD	0.44 (0.28-0.69)	<0.001	0.79 (0.49-1.30)	NS
Donor sex	0.72 (0.52-1.01)	NS	0.95 (0.64-1.42)	NS
Sex mismatch	0.66 (0.47-0.93)	0.019	0.87 (0.55-1.38)	NS
Recipient sex	0.66 (0.46-0.95)	0.026	0.76 (0.48-1.20)	NS
MELD	1.19 (0.96-1.20)	0.048	1.18 (0.96-1.99)	0.042
Recipient age	1.01 (0.99-1.03)	NS	1.01 (0.99-1.03)	NS
Recipient BMI	1.00 (0.99-1.02)	NS	1.00 (0.99-1.01)	NS

BMI, body mass index; CI, confidence interval; DBD, donation after brain death; DCD, donation after circulatory death; HR, hazard ratio; LD, living donor; MELD, Model for End-Stage Liver Disease; NS, not significant.

**FIGURE 3.**
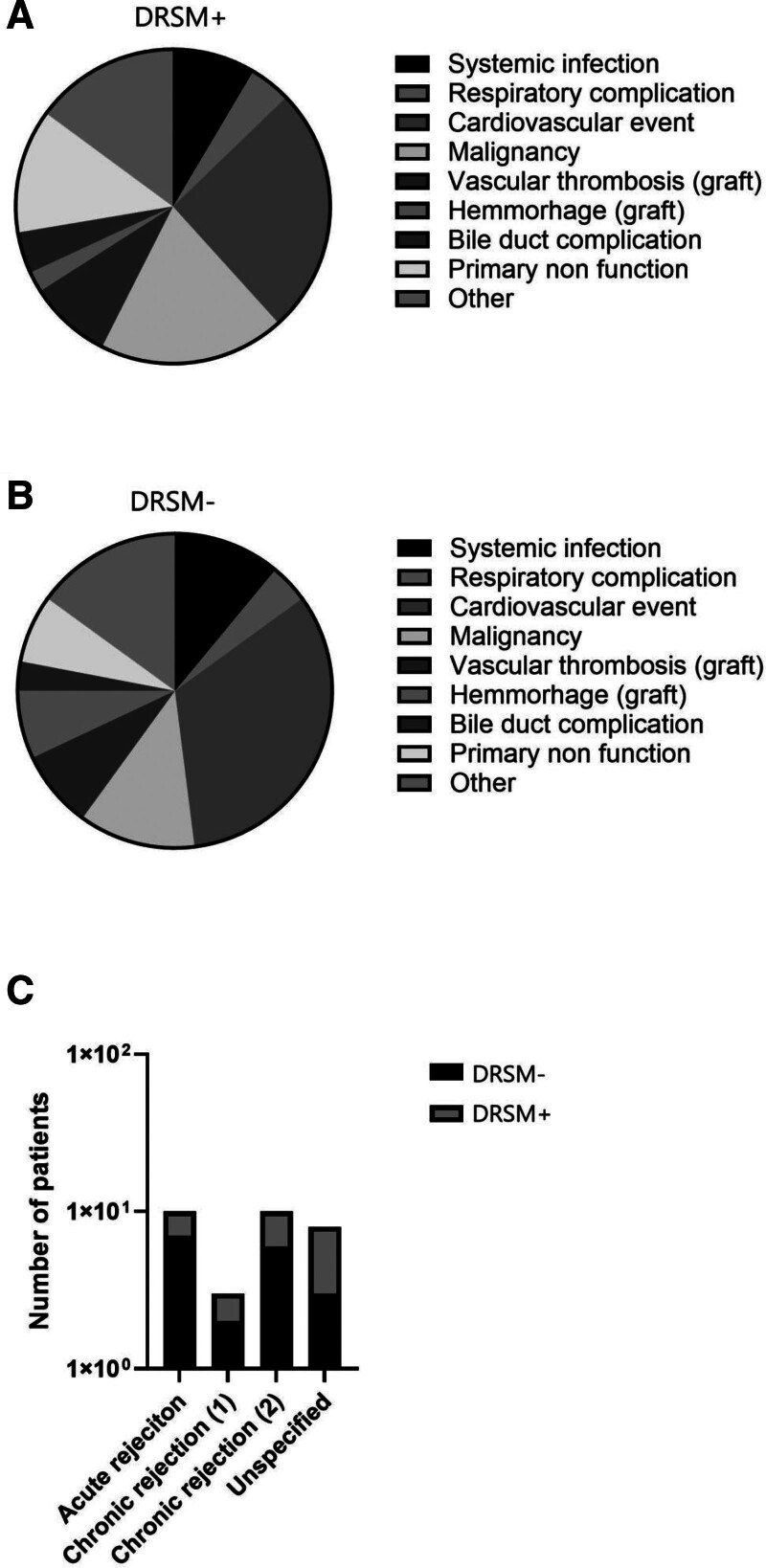
Cause of death within the patient cohorts. DRSM+ and DRSM− are compared in (A and B), respectively. Causes of death in the DRSM+ and DRSM− groups are shown in (A and B), respectively. C, Compares the overall acute rejection rate between the DRSM+ and DRSM− groups. DRSM, donor-recipient sex mismatch.

In both the DRSM+ and DRSM– groups, the predominant causes of death were comparable, with cardiovascular events—defined as myocardial infarction, sudden circulatory death, or stroke—representing the leading cause and accounting for 26% and 33% of all deaths, respectively (Figure 3). The second most prevalent cause of mortality among both groups was the reappearance of malignancies, including hepatocellular carcinoma and colorectal metastases, with a prevalence of 19% in the DRSM+ group and 12% in the DRSM– group. The mortality rate attributable to hepatic graft dysfunction was low in both the DRSM+ and DRSM– groups.

Vascular thrombosis, predominantly in the hepatic artery, and disseminated hemorrhage because of hepatic failure ultimately resulted in graft-related mortality. The incidence of graft rejection was minimal in both groups at a rate of 3% (Figure 3A and B). Acute graft rejection occurred with a higher frequency in the DRSM– group (39%) than in the DRSM+ group (23%; Figure 3C). Nevertheless, the findings did not reach statistical significance with respect to the cause of death and grade of rejection. In contrast, a higher percentage of patients in the DRSM+ group exhibited fatal outcomes because of primary nonfunction (PNF), with 13% and 7% of cases, respectively (Figure 3A and B).

### Evaluation of Risk Factors for Sex-specific Survival

The analysis of the entire study population indicated that elevated donor age (HR, 1.01; 95% CI, 1.00-1.03; *P* = 0.030) and elevated recipient MELD (HR, 0.98; 95% CI, 0.96-1.00; *P* = 0.042) were associated with a marked increase in mortality risk, as determined by univariable Cox regression analysis (Table 2). In addition, living donation was linked to a significantly decreased mortality risk in the univariable Cox regression analysis (HR, 0.44; 95% CI, 0.28-0.69; *P* < 0.001). Recipients with DRSM and female recipients demonstrated a significantly decreased mortality risk in the univariable analysis (DRSM+ recipients: HR, 0.66; 95% CI, 0.47-0.93; *P* = 0.019 and female recipients: HR, 0.66; 95% CI, 0.46-0.95; *P* = 0.026). However, DRSM was not identified as a significant factor affecting survival in the multivariate Cox regression analysis. The 2 factors of donor age and MELD were confirmed using multivariable Cox regression analysis; however, no significant differences were observed in the multivariable analysis.

### Sex-specific Survival and Donor Type-stratified Analyses

**FIGURE 4.**
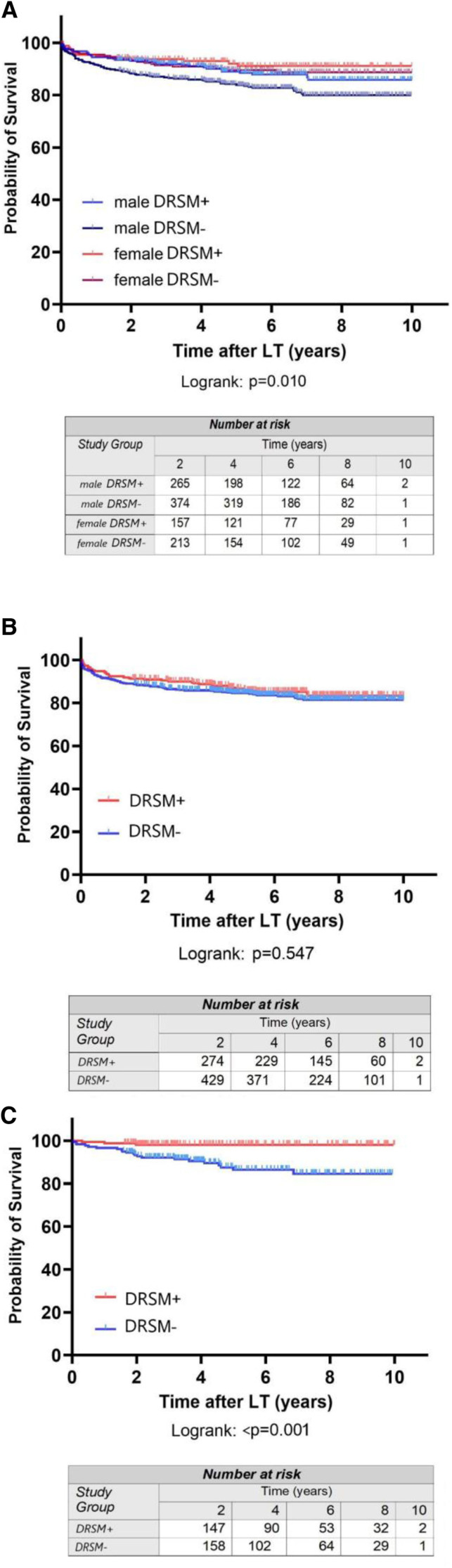
Survival analyses according to donor-recipient sex mismatch status in liver transplantation recipients. A, Survival subgroup analysis of sex-specific survival. B, Comparison of deceased DRSM+ and DRSM– groups. C, Survival analysis comparing living DRSM+ and DRSM– groups. DRSM, donor-recipient sex mismatch; LT, liver transplantation.

A subgroup survival analysis was performed on male and female recipients, with and without DRSM, to assess the differences between the DRSM+ and DRSM– groups (Figure 4A). The findings showed that male recipients had lower overall survival compared with female recipients, irrespective of DRSM. Additionally, female DRSM+ recipients exhibited the highest overall survival probability, with female DRSM– recipients ranking second, male DRSM+ recipients ranking third, and male DRSM– recipients ranking last. These results were statistically significant (*P* < 0.01).

Moreover, a statistically significant difference in donor type was observed between the DRSM+ and DRSM– groups (*P* = 0.016). The DRSM– group had a higher proportion of DD transplants and a lower proportion of LD transplants compared with the DRSM+ group (DD: 73% versus 65%; LD: 27% versus 35%, respectively; Table 2).

Subsequently, subgroup analyses stratified by donor type were performed to account for potential differences between LD and DD transplantation (Figure 4B and C). Among recipients of DD grafts, overall survival was comparable between DRSM+ and DRSM– groups (log-rank *P* = 0.547; Figure 4B). In contrast, within the LD cohort, a significant difference in survival was observed (log-rank *P* < 0.001), with higher survival in the DRSM+ group (Figure 4C).

## DISCUSSION

In this cohort of LT recipients from a high-volume center, DRSM was not associated with impaired long-term survival following LT. Although the unadjusted Kaplan-Meier analysis suggested slightly improved survival in the mismatched group, this association was not confirmed after adjustment using multivariable Cox regression and propensity score matching, indicating that sex mismatch itself is unlikely to represent an independent determinant of posttransplant survival. Therefore, the apparent survival advantage observed in the unadjusted analysis likely reflects differences in donor and recipient characteristics between groups, particularly the higher proportion of LDs and female donors in this cohort. After adjustment for these factors using multivariable analysis and propensity score matching, the apparent survival advantage of the DRSM+ group was no longer observed.

The role of DRSM in LT outcomes has been investigated from multiple perspectives, yet the available evidence remains heterogeneous. Earlier studies suggested that specific donor-recipient sex combinations, particularly female-to-male transplantation, might be associated with inferior graft survival, whereas more recent analyses have questioned whether these associations persist after adjustment for donor and recipient characteristics.^9,11,15^ At the same time, several donor and recipient characteristics are known to influence survival after LT, including characteristics that may be associated with DRSM, such as graft size and graft quality, which highlights that the clinical relevance of DRSM remains debated.^16,17^ In this context, our findings contribute to the interpretation of these discrepancies by providing a clinically granular, contemporary cohort analysis in which the apparent survival advantage observed in unadjusted analyses was no longer present after accounting for donor type and graft-related factors.

These findings are consistent with several previous studies suggesting that the influence of DRSM may be less pronounced than previously assumed.^9,11,15^ More recently, large registry-based, multicenter analyses of the United Network for Organ Sharing and Eurotransplant cohorts reported that sex-related differences in transplantation outcomes may exist but are often attenuated after adjustment for donor and recipient characteristics.^5,13^ These analyses highlight the complex and multifactorial nature of sex-related effects in LT outcomes.^18^

Within this framework, additional factors may also explain discrepancies between studies. In our cohort, the proportion of female donors and LD was higher in the sex-mismatched group than in the matched group. Changes in donor demographics, transplantation practices, and recipient characteristics over time may therefore influence the observed associations between sex mismatch and transplant outcomes.^4,10^ The interaction between donor type and sex mismatch has been highlighted in previous studies, suggesting differential effects depending on the type of donation.^10^ In line with this, our donor type-stratified subgroup analyses demonstrated that the association between sex mismatch and survival differed between LD and DD transplantation. While no significant effect of sex mismatch on survival was observed in DD transplantation, a survival difference was identified within the LD cohort. Notably, recipients in the sex-mismatched LD group showed improved survival compared with matched recipients. However, given the potential for residual confounding within subgroup analyses, these findings should be interpreted with caution. Although the number of male and female recipients in the LD group was balanced, further analyses would be required to determine whether specific mismatch combinations contributed to this observation.

From a clinical perspective, DRSM is not typically used as an isolated determinant when deciding whether to accept a liver graft or proceed with transplantation. Instead, organ acceptance decisions are primarily driven by factors such as donor age, graft quality, graft size, ischemia time, and recipient disease severity.^2,17^ However, sex mismatch may be associated with clinically relevant factors in certain situations. For example, differences in body size and liver volume between male and female donors may affect graft size matching, particularly when smaller female donor grafts are considered for larger male recipients.^2,3^ Several recent registry and cohort studies have suggested that apparent sex-related differences in transplantation outcomes may largely reflect differences in donor and recipient characteristics across study cohorts rather than sex mismatch itself.^5,13,19^ In line with these observations, our findings support the view that DRSM alone is unlikely to represent a decisive factor for donor selection in contemporary LT practice.

Consistent with previous reports, female recipients in our study demonstrated significantly improved long-term survival compared with male recipients.^10,18^ Several biological and immunological mechanisms have been proposed to explain these differences, including sex-related variations in immune responses, hormonal influences, and differences in the cause of underlying liver disease.^4^ Lifestyle factors and comorbidity profiles may also contribute to the observed survival advantage in female recipients.^20^ Conversely, some reports have suggested that female sex or DRSM may negatively influence outcomes because of higher levels of preformed HLA antibodies or hormonal differences.^21,22^ These conflicting observations highlight the complex and multifactorial nature of sex-related differences in LT outcomes and the importance of periodically reevaluating using contemporary cohorts.

Importantly, modern liver allocation systems are designed to prioritize medical urgency and graft compatibility rather than donor-recipient sex constellation. Consequently, sex mismatch is rarely considered as an isolated factor during graft acceptance decisions and is typically interpreted in the context of clinically relevant factors such as graft size, donor quality, and recipient characteristics. Our multivariable analysis confirmed that donor age and recipient MELD score were significant predictors of survival after LT, consistent with previous literature. In contrast, DRSM was not identified as an independent risk factor in adjusted models. Therefore, the higher proportion of LD transplantations and female donors in the DRSM+ group likely contributed to the improved survival observed in the unadjusted analysis.

Several limitations of this study should be acknowledged. The retrospective design inherently limits causal interpretation. In line with this, the statistically significant differences observed in unadjusted Kaplan-Meier analysis were not maintained after multivariable regression or propensity score matching. Collectively, both adjusted and unadjusted analyses consistently indicated that sex mismatch did not adversely affect long-term survival. Importantly, these proportions refer to the distribution of causes of death rather than the incidence of PNF in the entire cohort, and the absolute number of PNF events remained low in both groups. However, the relative difference warrants careful interpretation given the clinical impact of PNF on postoperative outcomes following LT. PNF is primarily driven by graft-related factors such as ischemia-reperfusion injury, donor quality, or perioperative conditions and is therefore best interpreted as a multifactorial event occurring early after transplantation.^23^ Notably, residual confounding related to donor and recipient characteristics may persist between groups, including factors such as graft quality and recipient clinical status at transplantation, which are known to influence early graft function.^17^ In addition, early graft function may have been influenced by current patterns in LT, including the persistent shortage of donor organs.^1^ In line with this, the absence of a difference in adjusted long-term survival suggests that this observation does not reflect a sustained effect of DRSM on transplant outcomes. Strengths of the present study include the relatively large contemporary cohort, the long follow-up period, and the availability of detailed donor and recipient characteristics from a high-volume transplant center.

In conclusion, our findings suggest that DRSM does not independently influence long-term survival after LT in contemporary clinical practice. Instead, donor and recipient characteristics such as donor age, graft quality, and recipient disease severity appear to play a more relevant role in determining outcomes. These results support the view that DRSM alone should not be considered a major limiting factor in donor selection.
